# Management of Atopic Dermatitis: The Role of Tacrolimus

**DOI:** 10.7759/cureus.28130

**Published:** 2022-08-18

**Authors:** Badar Uddin Umar, Sayeeda Rahman, Siddhartha Dutta, Tariqul Islam, Nadia Nusrat, Kona Chowdhury, Wan Farizatul Shima Binti Wan Ahmad Fakuradzi, Mainul Haque

**Affiliations:** 1 Medical Education, Oxford College of Arts, Business and Technology, Toronto, CAN; 2 School of Medicine, American University of Integrative Sciences, Bridgetown, BRB; 3 Pharmacology, All India Institute of Medical Sciences, Rajkot, Rajkot, IND; 4 Community Medicine, Gonoshasthaya Samaj Vittik Medical College, Dhaka, BGD; 5 Pediatrics, Delta Medical College & Hospital, Dhaka, BGD; 6 Pediatrics, Gonoshasthaya Samaj Vittik Medical College, Dhaka, BGD; 7 Community Medicine, Faculty of Medicine and Defence Health, Universiti Pertahanan Nasional Malaysia (National Defence University of Malaysia), Kuala Lumpur, MYS; 8 Pharmacology and Therapeutics, National Defence University of Malaysia, Kuala Lumpur, MYS

**Keywords:** corticosteroid medication, tacrolimus, capacity, function, atopic dermatitis, pharmacological interventions

## Abstract

Atopic dermatitis (AD) is a long-lasting inflammatory dermatological condition characterized by itchy, eczematous, sparsely tiny blisters that hold a clear watery substance. Additionally, the diseased skin can suppurate, occasionally with weeping with thickening of the affected skin. This is considered one of the top skin disorders involving both children and adult populations globally. The principal therapeutic intervention for AD is long-standing topical glucocorticoids, which have been used for several decades. Corticosteroid therapy brings several adverse drug effects (ADRs), including irreversible skin atrophy. Tacrolimus belongs to the class of calcineurin inhibitors, which is a type of immunomodulator possessing promising efficacy in treating AD. Topical tacrolimus is an effective and safe non-corticosteroid substitute treatment for AD. We reviewed the available literature to compare and institute the safety, efficacy, and effectiveness of tacrolimus when equated to corticosteroid therapy in managing AD.

## Introduction and background

Tacrolimus is a very potent anti-T-lymphocyte, macrolide, and immunosuppressant medicine produced from the fungus* Streptomyces tsukubaensis*; it was discovered in 1984 [1-3]. It has been reported that the therapeutic interventions for atopic dermatitis (AD) have vastly changed after the advent of tacrolimus [4]. The topical application of tacrolimus at a concentration of 0.03% to 0.1% has been reported to possess effectiveness therapeutically among pediatric (middle childhood [2-6 years] and school aged [7-15 years] children) and adult patients [4-6]. A lower concentration (0.03%) in the pediatric age cluster is recommended and is contraindicated in the usage below two years of age [7].

Diverse role of tacrolimus as an immunomodulator in transplant and dermatology

The concept of substituting poorly or not functioning vital body parts or tissues with physiologically healthy (homologous) ones is an old concept [8,9]. Nevertheless, in 1954, the first effective and efficient human renal transplant was achieved [9]. In the last 70 years, there has been a massive improvement in transplant-related surgical methods, instruments, and organ conservation [9-12]. Comprehensive pathophysiological knowledge regarding immunologic barriers to transplantation has improved immensely. [12,13] The progress of novel immunosuppressive agents with higher potent effects was the principal driving force for the human organ transplantation routine [8,14,15]. The current success of transplantation and prolonging human life is often notified because of tacrolimus (FR000506/FK506) [9,16-19]. Some multicentered studies demonstrated therapeutic safety and efficiency of systemic and topical use of tacrolimus in common inflammatory skin diseases such as psoriasis and AD, with only a few local side effects [20].

Atopic dermatitis

Brief Overview Atopic Dermatitis

AD is a long-lasting, reverting, exceedingly pruritic inflammatory condition, and one of the top dermatological disorders [21-23]. AD is considered a specific form of eczema causing intense itching and considerable sleep disorders and often promotes social stigma and increases emotional and psychological stress [24-28]. This dermatological disease significantly increases morbidity and destructively disturbs the patient’s and family’s quality of life [29-33]. To date, the pathology of the disease remains obscure. Nevertheless, it involves an abnormally functioning immune system, genetic predisposition, epidermal gene metamorphosis, and atmospheric aspects that instigate the disruption of the epidermal physiology and promote the development of AD [34-39], and it is frequently found challenging to relieve these patients [40]. The management of this dermatological disorder is expensive among Asian communities, thereby increasing the disease burden and financial overhead both at the level of individual and society [41-43]. In the USA, the annual financial overhead cost of AD was US$5.297 billion in 2015 through conventional appraisal [43].

AD is a systemic disease [44] categorized by anomalous epidermal wall physiology [45], and it can spread over various parts of the body with multiorgan involvement [46]. The origin of epidermal wall interruption is composite and determined by multiple triggering features including structural, family inherited, environs, and immunological aspects [34,47-49]. Furthermore, epidermal and gut microbiota modification often influences AD’s disease severity, length, and poor response to therapeutic intervention [50,51]. Clinically, AD can make headway from dermatological disorders to food hypersensitivity, hay fever, and later bronchial asthma, frequently recognized as the atopic march [52,53]. Additionally, multiple studies have reported that AD has the potential to develop systemic inflammatory diseases involving gastrointestinal or airway disorders. Nevertheless, these findings have not yet been verified scientifically [44,54,55].

Glucocorticoid in Atopic Dermatitis

Glucocorticoids directly applied to the affected part of the skin remain the backbone of AD management over the previous 60 years [56-58]. Hydrocortisone was identified as the initial steroid cast-off in AD [56-58]. Later, the world's regulatory bodies approved 30 glucocorticoid molecules with diverse potency for treating AD [56]. It has been reported that topical glucocorticoids are recurrently irrationally prescribed and consumed [59-61]. The long-standing use of dermal glucocorticoids is associated with adverse drug reactions (ADRs) [62]. The reported ADRs are hooked on the biochemical properties of the drug, the vehicle, and at the site of medicine applied [63]. ADRs such as atrophy, striae, rosacea, perioral dermatitis, acne, and purpura are repeatedly reported. Hypertrichosis, pigmentation remodeling, retard traumatic lesion to reinvigorate process, and exasperation of dermatological infections are also low in frequency [59,60,63-65]. Therefore, it has been advised that even topical glucocorticoid administration should be carefully monitored to minimize ADRs [61,62]. Doctors and nurses should counsel the patients on applying topical high-potency steroids to maximize the benefit and minimize ADRs [65-68]. High-potency dermatological corticosteroids must not be used for two to four weeks [60,63,69].

## Review

Tacrolimus: mode of action

Dermatological tacrolimus is a useful immunomodulator [70,71]. It attaches to specific cytoplasmic immunophilin (FKBP12), Ca2+, and calmodulin, hindering or suppressing calcineurin's phosphatase activity. This further impairs the nuclear factor of activated T-cells (NFAT) signaling process by preventing its dephosphorylation, which cumbers upon interleukin-2 (IL-2) generation and results in diminished T-cell activation and inflammatory cytokine let out [72-74]. Topical tacrolimus reduces mononuclear cell infiltration, thereby partly bringing back hair growth and hence can be valuable in alopecia areata [20,75,76]. It is also reported that tacrolimus impedes the conglomerate of IL-2, IL-3, IL-4, tumor necrosis factor-alpha (TNF-α), and granulocyte-macrophage colony-stimulating factor (GM-CSF) [1]. Apart from these, there is also evidence that tacrolimus hinders the let-out pre-formed inflammatory molecules from mast cells and basophils present in the skin layers and minimizes the utterance of FcεRI on Langerhans cells [77,78].

Topical tacrolimus is helpful for several inflammatory skin disorders, including vitiligo, psoriasis, alopecia areata, contact allergy, lichen planus, pyoderma gangrenosum, ichthyosis linearis circumflexa, and skin grafting/transplant [4,79-81].

Advantage of tacrolimus over corticosteroids

Efficacy and Effectiveness

Various studies have demonstrated the benefits and ADRs of tacrolimus in contrast to either vehicle base or diverse potencies of dermal glucocorticoids. Several clinical studies have established tacrolimus's relative efficacy (Table 1) and safety over corticosteroids [82-89]. There is abundant evidence suggesting the safety and efficacy of tacrolimus and other calcineurin inhibitors over long-term topical corticosteroid therapy for AD and other dermatological conditions [84,90-93]. The beneficial and advantageous effects of tacrolimus were found in pediatric and adult patients with AD (Table 1) [94].

**Table 1:** Studies showing a comparison between tacrolimus (calcineurin inhibitors) and corticosteroids AD, atopic dermatitis; AE, adverse effect

	Authors	Year	Study Type: Disease	Drugs Used	Result/Findings
1	Mandelin et al. [84]	2010	RCT: AD	Tacrolimus ointment. Hydrocortisone acetate 1%. Hydrocortisone butyrate 0.1%	Tacrolimus was found to have superior efficacy over steroids. The AE profile of corticosteroids was a little better, not statistically significant though (40/40 vs. 34/40, respectively.
2	Axon et al. [85]	2021	Umbrella review: AD	Topical corticosteroids. Topical calcineurin inhibitors. Vehicles/ emollients	There was a higher relative risk of skin thinning with topical corticosteroids. Biochemical adrenal suppression was evident with corticosteroids.
3	Tabędź and Pawliczak R. [86]	2019	Meta-analysis of RCTs: AD	Tacrolimus (0.3%, 0.1%). Pimecrolimus (1%). Glucocorticoids	Calcineurin inhibitors were significantly more effective. Skin burning and pruritus were the common AEs.
4	Koh et al. [87]	2021	Retrospective review of medical records: ocular surface inflammation in pediatric patients	Topical tacrolimus (0.02%). Topical corticosteroids	48% of patients recovered fully before 12 months, and 56% continued 12 months of therapy. All patients who continued showed improvement with tacrolimus. AEs were more familiar with corticosteroids.
5	Ohtsuki et al. [83]	2018	Review: AD	Topical tacrolimus. Topical corticosteroids	Tacrolimus is effective and well tolerated in the long-term treatment and can improve quality of life. There is no current strong evidence of an increased malignancy in risks. Data from post-marketing surveillance show no safety concerns.
6	Nakagawa [82]	2006	Review of randomized, double-blind clinical studies: AD	Topical tacrolimus 0.1%. Topical corticosteroids	Tacrolimus is superior to mild potency corticosteroids in both efficacy and safety. Tacrolimus is safe in long-term use too.
7	Svensson et al. [94]	2011	A systematic review of tacrolimus ointment compared with corticosteroids: AD	Tacrolimus 0.1% ointment. Class I, II, and II corticosteroids	Tacrolimus and topical corticosteroids are effective in AD in children and adults.

Multiple studies observed that individuals with tolerable to grave AD showed noticeable more overall global improvement with topical tacrolimus (0.1% and 0.03%) than with the mild-to-moderate potent dermatological glucocorticoid after seven days [95-98]. Local application of tacrolimus was found to be efficacious and had low-profile ADRs, and thus is considered mainstream medication for AD patients [99]. The most common ADRs with tacrolimus were local irritations at the application site, generally resolved with continual treatment. The findings suggested that dermal tacrolimus is an effective, safe, non-corticosteroid substitute treatment for AD [82]. A study compared 0.1% topical use of tacrolimus with glucocorticoids. The tacrolimus and its’ congeners significantly improved the affected body surface area (p<0.001), higher than the steroidal cluster [98]. It was assessed through a modified eczematous area and severity index (mEASI), eczematous area, and severity index (EASI). The tacrolimus cluster suffered a higher rate (P<0.001) of a smoldering commotion than the corticosteroids. However, the skin burning resolved in one week, and no infection or malignancies were reported [98]. Topical tacrolimus 0.03% has been found as more efficacious and safer than mild corticosteroids. Besides AD, tacrolimus also showed promising efficacy in other conditions, including oral lichen planus and labial discoid lupus erythematosus [100,101].

Safety

Adverse effect profiles of both long-term and short-term corticosteroids are more serious than tacrolimus [102]. The most frequent adverse effect of topical tacrolimus includes a burning sensation on the skin or irritation. However, burning skin-related adverse effects most frequently appear in the early part of the pharmacological intervention and usually exist for short periods. It has been reported that skin irritation stops within the first few days of therapy [103]. Topical tacrolimus does not cause skin atrophy, which occurs during the treatment with topical corticosteroids [104]. Tacrolimus dermatological preparation was not associated with an increased risk of cutaneous infections (bacterial and viral infections), which usually coexist with AD [105-108]. Other cutaneous infections found due to treatment with tacrolimus include herpes simplex, varicella, and eczema herpeticum [108,109]. Many studies have warned about the potential risk of developing cancer (mainly lymphoma and skin cancer) [110-112]. However, other research revealed that the use of topical calcineurin inhibitors is not grounded on long-standing safety data and the information derived from animal studies [91,113,114]. However, studies do not reveal that tacrolimus raises the risk of lymphoma and melanoma skin cancer [82,91,110,115-119]. Long-standing safety of dermal tacrolimus was observed in trials for up to four years [84]. A study on healthy volunteers demonstrated that tacrolimus causes almost no functional changes to healthy skin due to its poor permeability compared to topical corticosteroids [120].

The topical use of corticosteroids may cause skin atrophy; however, there was no reported possibility of malignancies or skin atrophy due to tacrolimus [83,97]. Tacrolimus effectively treats moderate-to-severe AD without causing atrophy due to prolonged use of topical corticosteroids [121].

## Conclusions

AD is a continuing inflammatory dermal disease that negatively impacts the patient's quality of life. It affects both children and adults. The treatment is usually long-term and often unsatisfactory on account of the ADRs and the possibility of relapse. There has been a continuous search for a safer, efficacious therapy for long-term remission. Among the available corticosteroids, calcineurin inhibitors, emollients, antihistamines, and immunosuppressants are mentioned. This review intended to explore the efficacy and safety of tacrolimus over topical steroids. It was evident from the available trials, systematic reviews, and other studies that tacrolimus is equi-efficacious to glucocorticoids in ameliorating AD symptoms. Except for skin irritation or burning sensation, the safety profile of tacrolimus is similar to that of corticosteroids. Appropriate patient education can help patients and families overcome the fear of AD therapy. Large-scale clinical trials, systematic reviews, and meta-analyses could improve comprehension knowledge regarding the safety and efficacy of tacrolimus. This can motivate drug regulatory agencies to review the drugs' safety warnings.
